# A gut check: understanding the interplay of the gastrointestinal microbiome and the developing immune system towards the goal of pediatric HIV remission

**DOI:** 10.1186/s12977-024-00648-9

**Published:** 2024-10-18

**Authors:** Nicole Soo, Omotayo Farinre, Ann Chahroudi, Saikat Boliar, Ria Goswami

**Affiliations:** 1 Department of Pediatrics, Weill Cornell Medicine, New York, NY 10021 USA; 2 Department of Pediatrics, Emory University School of Medicine, Atlanta, GA 30322 USA; 3 Center for Childhood Infections and Vaccines of Children’s Healthcare of Atlanta and Emory University, Atlanta, GA 30322 USA; 4 Department of Microbiology and Immunology, College of Veterinary Medicine, Cornell University, Ithaca, NY 14853 USA; 5 Gale and Ira Drukier Institute for Children’s Health, Weill Cornell Medicine, New York, NY 10021 USA; 6 Present Address: Department of Microbiology and Immunology, Jacobs School of Medicine and Biomedical Sciences, University at Buffalo, Buffalo, NY 14203 USA

**Keywords:** Children living with HIV, Gastrointestinal microbiome, Immune ontogeny, ART-free HIV remission, Microbiome modulation

## Abstract

Despite the efficacy of antiretroviral therapy (ART) in reducing the global incidence of vertical HIV transmissions, more than 120,000 children are still infected with the virus each year. Since ART cannot clear the HIV reservoir that is established soon after infection, children living with HIV (CLWH) are forced to rely on therapy for their lives and suffer from long-term drug-related complications. Pediatric HIV infection, like adult infection, is associated with gut microbial dysbiosis, loss of gut epithelial integrity, bacterial translocation, CD4 + T cell depletion, systemic immune activation, and viral reservoir establishment. However, unlike in adults, HIV that is vertically acquired by infants interacts with a gut microbiome that is continuously evolving while concomitantly shaping the infant’s immune ontogeny. Therefore, to determine whether there may be interventions that target the HIV reservoir through microbiome-directed approaches, understanding the complex tripartite interactions between the transmitted HIV, the maturing gut microbiome, and the developing immune system during early life is crucial. Importantly, early life is the time when the gut microbiome of an individual is highly dynamic, and this temporal development of the gut microbiome plays a crucial role in educating the maturing immune system of a child. Therefore, manipulation of the gut microbiome of CLWH to a phenotype that can reduce HIV persistence by fostering an antiviral immune system might be an opportune strategy to achieve ART-free viral suppression in CLWH. This review summarizes the current state of knowledge on the vertical transmission of HIV, the developing gut microbiome of CLWH, and the immune landscape of pediatric elite controllers, and explores the prospect of employing microbial modulation as a potential therapeutic approach to achieve ART-free viral suppression in the pediatric population.

## Background

Despite the widespread availability and efficacy of antiretroviral therapy (ART) in reducing vertical HIV transmissions [1], pediatric HIV continues to be a major global health concern, with nearly 120,000 children infected with the virus annually [2]. As a result, approximately 1.4 million children worldwide were living with the virus, in 2023 [2]. While ART is efficacious in suppressing productive HIV replication and controlling HIV-associated disease progression, it cannot eliminate the viral reservoir that is established soon after infection. Consequently, to keep the productively replicating virus suppressed, children living with HIV (CLWH) are forced to adhere to ART for their entire lives. Life-long ART is associated with huge financial burden [3], social stigma [4] and complications such as the development of drug-resistant viral variants [5], and long-term metabolic and neurologic diseases [6]. These limitations highlight the necessity for the development of safe and cost-effective novel approaches to achieve drug-free viral suppression in the pediatric population.

Colonization of the gastrointestinal (GI) tract by microbes begins at parturition and/or immediately after birth (though some have proposed potential colonization even in utero [7–9]) and continues to evolve during the first three years of life. The evolution of early life microbiome is dependent on multiple factors such as birth mode, gender, maternal microbial composition, diet (breast milk or formula), antibiotic exposure, geographical locations, and environmental factors [10]. The maturing microbiome plays a crucial role in shaping a child’s immune development [11] and has been increasingly recognized as a contributor to human disease [12] including infectious diseases [13]. Notably, the first 2 years of life is also the time when children are exposed to HIV both perinatally (in utero and during delivery) and postnatally (during breastfeeding). Pediatric HIV infection is associated with alterations in the gut microbial composition [14, 15], massive depletion of CD4 + T cells in the GI tract [16], inflammation and immune activation [17], and establishment of viral reservoirs [18]. Therefore, to identify strategies that can reduce viral reservoir size and achieve viral remission in the absence of ART, delineating the interactions of gut resident microbes with the maturing immune system of a child living with HIV will be key. This review will summarize our current knowledge of the trans-kingdom interactions between the vertically-transmitted HIV and gut microbiome with the maturing immune system of CLWH. Additionally, we will focus on interventions targeting the microbiome with implications for drug-free HIV remission in the pediatric population.

## Main text

### HIV persistence in the pediatric population

The majority of children who are infected perinatally or postnatally through breastfeeding, if left untreated, succumb to AIDS within two to three years of age [19]. Initiation of ART in children results in suppression of plasma viral loads, with almost equal potency as that of adults [20, 21]. Early initiation and adherence to ART significantly impede disease progression and result in a relatively smaller HIV reservoir size [22–24], but do not eliminate the reservoir. This is evident from the case of “Mississippi baby” who was born with HIV and was put on ART within 30 h of birth [25]. After 18 months of ART, treatment was discontinued. Although this child remained virally-suppressed without ART for 27 months, HIV eventually rebounded, indicating very early seeding and persistence of rebound-competent HIV in therapy-suppressed children. Similar rapid rebound in viremia within 7 to 35 days of analytical treatment interruption has been documented in experimentally Simian/Human immunodeficiency virus (SHIV)-infected infant rhesus macaques [26, 27]. In contrast to adults, the rate of decay in proviral DNA levels is considerably slower in children [28] which might indicate either inherent differences in the nature of latently infected cells in adults and children, or an inefficient elimination of infected cells by the relatively immature immune system of children. Furthermore, clonal expansion of latently infected cells is considered a major mechanism for the maintenance of HIV reservoirs over time [29, 30]. Latently infected, resting CD4 + T cells isolated from vertically-infected children can be activated to produce replication-competent virus, confirming their potential to contribute to the viral recrudescence upon therapy cessation [31].

HIV persistence on ART in children is not very well-characterized due to the challenges of inadequate volume of blood samples available for quantitative assessments [32]. Unlike adults, where memory CD4 + T cells are the main cellular source of harbored HIV during ART [33–35], in children, naïve CD4 + T cells are a major contributor to the total HIV reservoir [27, 36]. Additionally, using SHIV-infected infant rhesus macaques, the GI tract was demonstrated as a major anatomical site that harbors viral RNA/DNA-positive T cells after long term ART-suppression [26, 37]. In these infant macaques, upon treatment interruption, gut resident CD4 + T cells were the source of the quickest and strongest viral rebound. Therefore, HIV persistence within cellular reservoirs in children represents a challenge that needs to be overcome to achieve ART-free viral control in the pediatric population.

### Gut microbiome in pediatric HIV infection

HIV exposure is associated with disruption of the gut epithelial barrier and translocation of GI microbes and microbial products to the lamina propria, and eventually to systemic circulation [38]. Microbial translocation is a key driver of immune activation and chronic inflammation that leads to HIV disease progression [38–40]. HIV exposure-induced gut mucosal damage provides favorable conditions for alterations in the composition of gut microbial communities, also known as microbial dysbiosis [41]. Multiple studies in adults have discussed microbial dysbiosis in the GI tract, including alterations in the diversity of bacteria [42], fungi [43, 44] and virome [45, 46]. To date, only a few studies have focused on microbial dysbiosis in children and have only studied the gut bacteriome [14, 15, 47–50].

Similar to adults [51], the gut microbiome of CLWH also demonstrates a lower bacterial richness and diversity with altered colonization of bacterial taxa compared to children without HIV [14, 49, 50]. While there are differences in specific taxa altered upon HIV infection among various studies, owing to alterations in geographical location, age, route of HIV exposure and potential differences in the microbial sequencing approach, most of the studies have demonstrated an increase in the relative abundance of bacteria associated with immune inflammation and activation and a decrease in bacteria associated with maintenance of gut permeability and integrity. For instance, using a cohort of perinatally infected CLWH from India, Kaur et al., demonstrated an elevated relative abundance of *Prevotella*, a bacterial taxon associated with upregulation of microbial translocation marker, soluble CD14 [52], and immune inflammation marker [53], interferon-gamma inducible protein 10 (IP-10) [49]. In the same cohort of children and additional cohorts from Zimbabwe, Spirochaetes and Corynebacterium, bacteria that promote immune inflammation [54, 55], were upregulated during chronic HIV infection [49, 50]. Finally, *Lachnospiraceae* [49, 50, 56] and *Clostridia* [14], bacterial taxa that can produce anti-inflammatory short chain fatty acids such as butyric acid [57] and can maintain gut barrier integrity [58] were found to be downregulated in CLWH on suppressive ART, compared to uninfected children. Whether this HIV-induced modulation of the gut microbiome creates an immune environment that provides a competitive advantage for establishment and maintenance of a stable reservoir needs further investigation. In fact, in a recent clinical trial in adults living with HIV, the Bacteroidales:Clostridiales ratio was inversely correlated with HIV reservoir size and viral control post-analytical ART interruption (ATI) [59], highlighting the potential contribution of the microbiome of an individual on their HIV persistence status. Furthermore, in an in vitro study, butyric acid producers that are downregulated in CLWH on ART were shown to induce viral reactivation from latency [60], further highlighting the complex interaction that exists between gut bacterial environment and the vertically transmitted virus in maintaining a stable viral reservoir. To date, the majority of pediatric studies to understand the impact of viral infection on the gut microbiome in CLWH have focused on cohorts from low- and middle-income countries (LMICs), where the prevalence of HIV is highest. Consequently, HIV-mediated gut microbial dysbiosis in US pediatric cohorts remains uncharacterized. A recent study comparing the fecal microbiome of adults from the US, Botswana, and Uganda has indicated that despite the occurrence of HIV-mediated gut microbial dysbiosis in individuals from each region, there was no similarity between altered bacterial taxa across geographical regions [61]. Since the phylogenetic composition of the gut microbiome of children from different geographical locations is distinct due to differences in diet, cultural and socioeconomic status [62], studies focused on profiling any potential effect of HIV-mediated gut microbial changes on established viral reservoirs in children from distinct geographical locations would be crucial to better understand the interactions of vertically acquired HIV with the gut microbiome.

Moreover, to date, studies in CLWH interrogating the interactions of the virus with the gut microbial taxa have not specifically categorized children into perinatally (in-utero or during delivery) or postnatally (breastfeeding) infected. Therefore, future studies to delineate the differences in microbial dysbiosis among perinatally and postnatally infected children should be performed.

While the interactions of the developing gut microbiome of CLWH with the establishing viral reservoir are investigated, the impact of small molecular antiretrovirals and common childhood antibiotics, that have the potency to alter the colonization of gut microbiome needs to be considered. Based on the recommendations from The Panel on Antiretroviral Therapy and Medical Management of Children Living with HIV, currently, ART is initiated on every child diagnosed with HIV [63]. Studies in CLWH have demonstrated that despite the administration of effective ART, alterations in gut microbiota composition persist and long-term ART only partially resolves gut microbial dysbiosis [14, 50]. However, ART can also potentially contribute to an altered microbiome [14, 49]. Moreover, exposure to multiple types of ART regimens such as Ritonavir-boosted protease inhibitor or non-nucleoside reverse transcriptase inhibitor (NNRTI)-based regimens resulted in differential microbial colonization [14]. Specifically, children on protease inhibitor-based regimens had lower bacterial diversity compared to those on NNRTI-based regimens. It is hypothesized that this protease inhibitor-mediated alteration of the gut microbial colonization is due to altered microbial metabolism, that inactivates cytochrome P450 [14]. Similar to ART, the effect of cotrimoxazole, a broad-spectrum antibiotic commonly prescribed in children of developing countries [64], has also been investigated in the setting of HIV infection [15, 50]. Antibiotic treatment resulted in the alteration of the gut microbiome of CLWH to a dampened inflammatory phenotype [65]. Furthermore, differential gut bacterial taxa were reported in CLWH treated with ART and cotrimoxazole compared to antibiotic-naive ART-treated children [15], implying interactions of antibiotics and antiretrovirals. Additionally, HIV-induced microbial dysbiosis in children might also lead to differential metabolism of antiretrovirals and antibiotics leading to reduced bioavailability of the drugs [66], although this hypothesis needs to be investigated in the setting of pediatric HIV infection.

### Impact of gut microbiome on the developing immune system in pediatric HIV infection

The immune system of pediatric elite controllers (PECs), an extremely rare group of children who can control HIV without ART [67, 68], provides a model to guide the development of therapeutic strategies to achieve drug-free long-term HIV suppression. An altered immune profile in PECs compared to pediatric progressors (PPs), a population that progresses to disease, in the absence of therapy, has been documented [68]. Since the gut microbiome in a child plays a crucial role in educating and shaping the immune system [69], we hypothesize that modulating this microbiome-driven early-life immune ontogeny to a landscape that mimics that of PECs might be a potential way to achieve ART-free viral suppression. To build an immune system that can control HIV replication without treatment, understanding the influence of specific gut microbial species on the immune response observed in PECs is key.

The acute phase of HIV infection is characterized by activation and dramatic depletion of CD4 + T cells. After ART initiation, CD4 + T cell levels are partially restored, active HIV replication is suppressed, and life-long viral latency is established in memory CD4 + T cells [70–72]. Unlike PPs, PECs have higher proportions of naïve CD4 + T cells and lower activated and exhausted memory CD4 + T cells [68]. Since multiple studies demonstrated that in the pediatric population, naïve and not memory CD4 + T cells are the major contributors to the total HIV reservoir [27, 36], understanding how gut commensal microbes interact with these two populations of CD4 + T cells will be important to determine the influence of gut microbiome on HIV persistence. Gut commensal bacteria can drive the differentiation of intestinal naïve CD4 + T cells to T cell effector subsets, including Th17 and regulatory T cells (Tregs). Importantly, *Faecalibacterium* was correlated with a higher abundance of naïve CD4 + T cells [73]. A proper balance of functional Th17 and regulatory T cells (Tregs), involved in the maintenance of gut mucosal integrity and dampened immune activation, was indicated to be crucial for HIV suppression in the pediatric population [74–76]. Interestingly, gut bacterial species including, *C. coccoides*, *Staphylococcus*, *C. perfringens* and *Bacteroides fragilis* predicted the levels of Tregs and Th17 cells in pediatric cohorts living with the virus [15]. On the other hand, gut bacterial commensals can interact with intestinal memory CD4 + T cells and drive their activation and release of epithelial barrier-protective cytokines [77]. While the association of CD4 + T cell activation status with HIV reservoir is lacking in PECs, in adult elite controllers, this T cell phenotype was associated with a reduced HIV reservoir size [78, 79]. Therefore, gut microbial components that can induce a CD4 + T cell phenotype as observed in PECs might lead to reduced viral persistence. Using multiple cohorts of vertically-infected CLWH, a higher relative abundance of gut bacterial taxa *Clostridium coccoides*, *Staphylococcus*, *Clostridium perfringens*, *Succinivibrionaceae,* and *Lachnospira* were associated with elevated counts of systemic CD4 + T cells [15, 50, 73].

While the influence of the gut microbiome on CD8 + T cell functionality in CLWH has not been characterized yet, taxa such as *Lachnospira* have been associated with an elevated frequency of CD8 + memory T cells [73]. Humoral immunity, especially B cell functionality and non-neutralizing antibody functions has been associated with improved HIV control in the pediatric population [80, 81]. However, the relationship between gut microbiome and HIV-specific humoral immune responses has not been characterized in CLWH. Owing to the relatively immature adaptive immune response in children, innate immune cell functionality is crucial for achieving HIV control. While studies characterizing innate immune cell functionality in PECs are currently lacking, these cells were shown to be influenced by gut microbial species. For instance, in a cohort of CLWH, *Ruminococcus* was negatively associated with the NK cell population [73]. Additionally, gut bacterial taxa such as *Lachnospira* and *Ruminococcus* dampened overall immune inflammation in CLWH [49, 73]. To determine whether these associations are indicative of any causal relationships, further intervention studies delineating the role of these commensal bacteria on the HIV reservoir in the pediatric population will be needed. Profiling the gut microbiome of PECs might also be crucial to understanding the role of commensals on HIV persistence. However, one should be cautious in interpreting the data, as viral control due to the pre-existent immune and genetic features in PECs [67, 68] might contribute to an altered gut microbial profile in this population. In this scenario, non-human primate models of viral infection where the temporal nature of gut microbial evolution and establishment of HIV reservoir can be studied in parallel could be instrumental.

### Microbial modulation as a potential therapy to achieve pediatric HIV remission

**Fig. 1:**
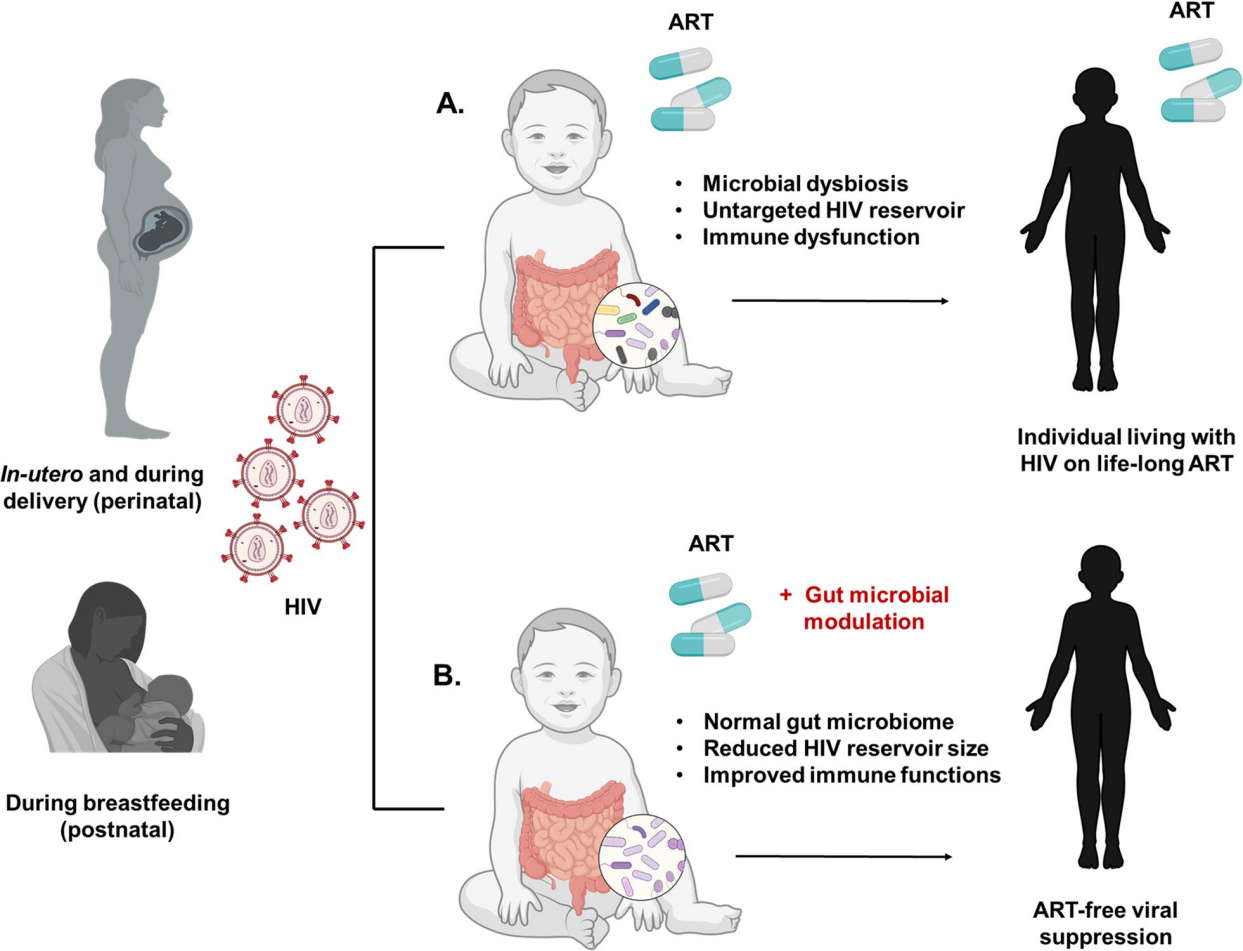
Gut Microbial modulation as a potential strategy to achieve ART-free HIV remission. Modulation of the gut microbiome of children who acquired HIV perinatally (in utero or during delivery) or postnatally (via breastfeeding) to a phenotype that reduces HIV reservoir size and repairs HIV-mediated immune imbalances might potentially lead to ART-free viral suppression in the pediatric population

The intestinal microbiome of children is most plastic during the first 2–3 years of life, when it can be shaped by external factors such as diet and environmental exposure. It is believed that the mother’s womb is mostly sterile and the first microbial seeding of an infant starts during birth. However, several groups have documented the possibility of microbial colonization in utero by demonstrating the presence of microbial communities in the placenta, amniotic fluid, and meconium [8, 82, 83]. As the baby descends through the birth canal, bacterial taxa such as *Lactobacilli* and *Bifidobacteria*, commonly found in the mother’s vaginal tract, colonize the baby’s intestine by entering through their mouth [84, 85]. Therefore, the mode of delivery of the baby is a crucial predictor of their gut microbial profile. Babies delivered by Cesarean section exhibit an abundance of bacterial taxa obtained from their mother’s skin and the environment, including *Clostridiodes difficile*, *Staphylococcus*, *Corynebacterium* and *Propionibacterium* [86, 87]. After parturition, an infant’s gut microbial community drastically develops and is influenced by factors such as skin-to-skin contact and breastfeeding vs. formula feeding. Compared to formula-fed, breastfed infants exhibit an abundance of *Lactobacillus* and *Bifidobacterium* species [88], which evolve to a *Bacteroides*- and *Firmicute*-rich phenotype upon introduction of solid food [89]. Upon weaning, a child’s gut microbiome rapidly undergoes maturation for the first 2–3 years of life, leading to a more stable adult-like microbiome [90]. Therefore, the period when the microbiome of a child is most dynamic might be the window of opportunity when microbiota-targeted interventions can modulate the child’s intestinal microbiome composition and build a metabolic and inflammatory condition that could potentially reduce HIV replication and persistence. Similar to adults living with HIV [91, 92], several gut microbiome-targeted approaches have been investigated in CLWH, with an aim to alter their gut microbiome and associated immune phenotypes. In a pilot placebo-controlled double-blinded study in CLWH [56], nutritional supplementation with PMT25341, a mixture of probiotics (*Saccharomyces boulardii*), prebiotics (long chain fructo-oligosaccharides, galacto-oligosaccharides) and supplements such as essential amino acids (arginine and glutamine), long chain fatty acids (eicopentaenoic and docosahexanoic acid), vitamin D and AM3, a glycopeptide produced by *Ricinus communis* was shown to restore the gut microbial imbalance caused by HIV infection, suggesting the feasibility of modulating the microbial composition in a pediatric population. The impact of this nutritional supplement on alterations of gut microbiome composition has also been shown in adults living with HIV [91], where the PMT25341 intervention group demonstrated enrichment of anti-inflammatory bacterial taxa that are depleted among CLWH [57]. However, this nutritional supplementation did not result in immune modulation among children [73]. In contrast, short-term oral probiotic therapy with milk containing bacterial species including *Bifidobacterium bifidum*, *Streptococcus thermophilus*, *Lactobacillus casei* Shirota (LcS), *Lactobacillus sporogens* and *Lactobacillus plantarum IS-10506* resulted in modulation of the maturing immune system of CWLH, including an increase in the frequency of peripheral CD4 + T, Th17 and Th2 cells, decrease in activated CD8 + T cells [93–95], and reductions of bacterial translocation marker, blood lipopolysaccharide (LPS) [96], and HIV plasma viral load [93]. Probiotics are clinically safe for most individuals [97], result in improved nutrition and growth in CLWH [93] and lead to persistent intestinal bacterial colonization with the administered organism [98]. In a pilot double-blind placebo-controlled study, oral capsular fecal microbial transplantation (FMT) of adults living with HIV demonstrated changes in the intestinal microbial composition to a phenotype observed in people without HIV and alterations in markers of intestinal injury [99, 100]. While FMT studies have not yet been conducted in CLWH, this gut modulation approach has been successfully applied for the treatment of several gastrointestinal diseases in children such as *Clostridiodes difficile* infection (CDI), ulcerative colitis and Crohn’s disease, where stool from healthy donor children of the same age and unrelated to the patient was used [101, 102]. This approach has been found to be safe in children [102]. In a retrospective study using 372 patients aged 11 months to 23 years, FMT to treat CDI was shown to be comparably safe and efficacious among children and young adults [103]. Taken together, these studies highlight the feasibility of modulating the gut microbiome of CLWH immediately after HIV diagnosis to a phenotype that might reduce HIV disease progression and interfere with HIV persistence by generating an anti-HIV immune system **(**Fig. 1**)**.

## Conclusions

The gut microbiome of an individual evolves and matures for the first 3 years of life, before achieving a stable adult-like phenotype. As the microbiome develops in a child, it also influences immune ontogeny. Therefore, understanding the interactions of the continuously evolving microbiome and maturing immunity in CLWH can be used to guide the development of strategies to achieve ART-free HIV control in the pediatric population. Here, we have reviewed the specific components of an early-life microbiome and immune responses in CLWH that might be exploited to reduce HIV persistence. Finally, we have discussed the currently used approaches of microbial modulation in this population that lead to altered immune phenotypes. Building a gut microbiome during early life that can reduce HIV persistence and improve anti-HIV immunity might be a promising approach to achieving ART-free long-term HIV control in the pediatric population.

## Data Availability

No datasets were generated or analysed during the current study.

## References

[1] Penazzato M, Kasirye I, Ruel T, Mukui I, Bekker A, Archary M, et al. Antiretroviral postnatal prophylaxis to prevent HIV vertical transmission: present and future strategies. J Int AIDS Soc. 2023;26(2): e26032. 36808699 10.1002/jia2.26032 PMC9939941

[2] UNAIDS. Fact sheet - Latest global and regional statistics on the status of the AIDS epidemic. 2023 https://www.unaids.org/en/resources/documents/2023/UNAIDS_FactSheet. Accessed 30 Sep 2024.

[3] Stelmach RD, Rabkin M, Abo K, Ahoba I, Gildas Anago M, Boccanera R, et al. Financial burdens of HIV and chronic disease on people living with HIV in Côte d’Ivoire: a cross-sectional out-of-pocket expenditure study. PLoS ONE. 2021;16(7): e0255074. 34324545 10.1371/journal.pone.0255074 PMC8320983

[4] Martinez J, Harper G, Carleton RA, Hosek S, Bojan K, Clum G, et al. The impact of stigma on medication adherence among HIV-positive adolescent and young adult females and the moderating effects of coping and satisfaction with health care. AIDS Patient Care STDS. 2012;26(2):108–15. 22149767 10.1089/apc.2011.0178 PMC3266519

[5] Van Dyke RB, Patel K, Kagan RM, Karalius B, Traite S, Meyer WA 3rd, et al. Antiretroviral drug resistance among children and youth in the united states with perinatal HIV. Clin Infect Dis. 2016;63(1):133–7. 27056398 10.1093/cid/ciw213 PMC4901868

[6] Leonard EG, McComsey GA. Metabolic complications of antiretroviral therapy in children. Pediatr Infect Dis J. 2003;22(1):77–84. 12544413 10.1097/00006454-200301000-00018

[7] Collado MC, Rautava S, Aakko J, Isolauri E, Salminen S. Human gut colonisation may be initiated in utero by distinct microbial communities in the placenta and amniotic fluid. Sci Rep. 2016;6(1):23129. 27001291 10.1038/srep23129 PMC4802384

[8] Jiménez E, Marín ML, Martín R, Odriozola JM, Olivares M, Xaus J, et al. Is meconium from healthy newborns actually sterile? Res Microbiol. 2008;159(3):187–93. 18281199 10.1016/j.resmic.2007.12.007

[9] Aagaard K, Ma J, Antony KM, Ganu R, Petrosino J, Versalovic J. The placenta harbors a unique microbiome. Sci Transl Med. 2014;6(237):237. 10.1126/scitranslmed.3008599 PMC4929217 24848255

[10] Vandenplas Y, Carnielli VP, Ksiazyk J, Luna MS, Migacheva N, Mosselmans JM, et al. Factors affecting early-life intestinal microbiota development. Nutrition. 2020;78: 110812. 32464473 10.1016/j.nut.2020.110812

[11] Sanidad KZ, Zeng MY. Neonatal gut microbiome and immunity. Curr Opin Microbiol. 2020;56:30–7. 32634598 10.1016/j.mib.2020.05.011 PMC8729197

[12] Vijay A, Valdes AM. Role of the gut microbiome in chronic diseases: a narrative review. Eur J Clin Nutr. 2022;76(4):489–501. 34584224 10.1038/s41430-021-00991-6 PMC8477631

[13] Maciel-Fiuza MF, Muller GC, Campos DMS, do Socorro Silva CP, Peruzzo J, Bonamigo RR, et al. Role of gut microbiota in infectious and inflammatory diseases. Front Microbiol. 2023;14:1098386. 37051522 10.3389/fmicb.2023.1098386 PMC10083300

[14] Abange WB, Martin C, Nanfack AJ, Yatchou LG, Nusbacher N, Nguedia CA, et al. Alteration of the gut fecal microbiome in children living with HIV on antiretroviral therapy in Yaounde, Cameroon. Sci Rep. 2021;11(1):7666. 33828220 10.1038/s41598-021-87368-8 PMC8027858

[15] Nguyen QT, Ishizaki A, Bi X, Matsuda K, Nguyen LV, Pham HV, et al. Alterations in children’s sub-dominant gut microbiota by HIV infection and anti-retroviral therapy. PLoS ONE. 2021;16(10): e0258226. 34634074 10.1371/journal.pone.0258226 PMC8504761

[16] Veazey RS. Intestinal CD4 depletion in HIV / SIV infection. Curr Immunol Rev. 2019;15(1):76–91. 31431807 10.2174/1573395514666180605083448 PMC6701936

[17] Machiavelli A, Duarte RTD, Pires MMS, Zárate-Bladés CR, Pinto AR. The impact of in utero HIV exposure on gut microbiota, inflammation, and microbial translocation. Gut Microbes. 2019;10(5):599–614. 30657007 10.1080/19490976.2018.1560768 PMC6748604

[18] Katusiime MG, Van Zyl GU, Cotton MF, Kearney MF. HIV-1 persistence in children during suppressive ART. Viruses. 2021. 10.3390/v13061134. 34204740 10.3390/v13061134 PMC8231535

[19] UNAIDS. Children and HIV- Fact Sheet 2016 https://www.unaids.org/sites/default/files/media_asset/FactSheet_Children_en.pdf. Accessed 10 May 2024.

[20] Melvin AJ, Rodrigo AG, Mohan KM, Lewis PA, Manns-Arcuino L, Coombs RW, et al. HIV-1 dynamics in children. J Acquir Immune Defic Syndr Hum Retrovirol. 1999;20(5):468–73. 10225229 10.1097/00042560-199904150-00009

[21] Luzuriaga K, Wu H, McManus M, Britto P, Borkowsky W, Burchett S, et al. Dynamics of human immunodeficiency virus type 1 replication in vertically infected infants. J Virol. 1999;73(1):362–7. 9847340 10.1128/jvi.73.1.362-367.1999 PMC103841

[22] Uprety P, Patel K, Karalius B, Ziemniak C, Chen YH, Brummel SS, et al. Human immunodeficiency virus type 1 DNA decay dynamics with early, long-term virologic control of perinatal infection. Clin Infect Dis. 2017;64(11):1471–8. 28329153 10.1093/cid/cix192 PMC5434384

[23] Martínez-Bonet M, Puertas MC, Fortuny C, Ouchi D, Mellado MJ, Rojo P, et al. Establishment and replenishment of the viral reservoir in perinatally HIV-1-infected children initiating very early antiretroviral therapy. Clin Infect Dis. 2015;61(7):1169–78. 26063721 10.1093/cid/civ456 PMC4560905

[24] Persaud D, Patel K, Karalius B, Rainwater-Lovett K, Ziemniak C, Ellis A, et al. Influence of age at virologic control on peripheral blood human immunodeficiency virus reservoir size and serostatus in perinatally infected adolescents. JAMA Pediatr. 2014;168(12):1138–46. 25286283 10.1001/jamapediatrics.2014.1560 PMC4324476

[25] Persaud D, Gay H, Ziemniak C, Chen YH, Piatak M Jr, Chun TW, et al. Absence of detectable HIV-1 viremia after treatment cessation in an infant. N Engl J Med. 2013;369(19):1828–35. 24152233 10.1056/NEJMoa1302976 PMC3954754

[26] Obregon-Perko V, Bricker KM, Mensah G, Uddin F, Rotolo L, Vanover D, et al. Dynamics and origin of rebound viremia in SHIV-infected infant macaques following interruption of long-term ART. JCI Insight. 2021. 10.1172/jci.insight.152526. 34699383 10.1172/jci.insight.152526 PMC8675190

[27] Goswami R, Nelson AN, Tu JJ, Dennis M, Feng L, Kumar A, et al. Analytical treatment interruption after short-term antiretroviral therapy in a postnatally simian-human immunodeficiency virus-infected infant rhesus macaque model. MBio. 2019. 10.1128/mBio.01971-19. 31488511 10.1128/mBio.01971-19 PMC6945967

[28] Persaud D, Ray SC, Kajdas J, Ahonkhai A, Siberry GK, Ferguson K, et al. Slow human immunodeficiency virus type 1 evolution in viral reservoirs in infants treated with effective antiretroviral therapy. AIDS Res Hum Retroviruses. 2007;23(3):381–90. 17411371 10.1089/aid.2006.0175

[29] Maldarelli F. HIV-infected cells are frequently clonally expanded after prolonged antiretroviral therapy: implications for HIV persistence. J Virus Erad. 2015;1(4):237–44. 27482422 10.1016/S2055-6640(20)30930-4 PMC4946654

[30] Wagner TA, McLaughlin S, Garg K, Cheung CY, Larsen BB, Styrchak S, et al. Proliferation of cells with HIV integrated into cancer genes contributes to persistent infection. Science. 2014;345(6196):570–3. 25011556 10.1126/science.1256304 PMC4230336

[31] Persaud D, Pierson T, Ruff C, Finzi D, Chadwick KR, Margolick JB, et al. A stable latent reservoir for HIV-1 in resting CD4(+) T lymphocytes in infected children. J Clin Invest. 2000;105(7):995–1003. 10749578 10.1172/JCI9006 PMC377486

[32] Khetan P, Liu Y, Dhummakupt A, Persaud D. Advances in pediatric HIV-1 cure therapies and reservoir assays. Viruses. 2022. 10.3390/v14122608. 36560612 10.3390/v14122608 PMC9787749

[33] Zerbato JM, Serrao E, Lenzi G, Kim B, Ambrose Z, Watkins SC, et al. Establishment and reversal of HIV-1 latency in naive and central memory CD4+ T cells In Vitro. J Virol. 2016;90(18):8059–73. 27356901 10.1128/JVI.00553-16 PMC5008097

[34] Zerbato JM, McMahon DK, Sobolewski MD, Mellors JW, Sluis-Cremer N. Naive CD4+ T cells harbor a large inducible reservoir of latent, replication-competent human immunodeficiency virus type 1. Clin Infect Dis. 2019;69(11):1919–25. 30753360 10.1093/cid/ciz108 PMC6853701

[35] Siliciano JD, Kajdas J, Finzi D, Quinn TC, Chadwick K, Margolick JB, et al. Long-term follow-up studies confirm the stability of the latent reservoir for HIV-1 in resting CD4+ T cells. Nat Med. 2003;9(6):727–8. 12754504 10.1038/nm880

[36] Mavigner M, Habib J, Deleage C, Rosen E, Mattingly C, Bricker K, et al. Simian immunodeficiency virus persistence in cellular and anatomic reservoirs in antiretroviral therapy-suppressed infant rhesus macaques. J Virol. 2018;92(18):10–128. 10.1128/JVI.00562-18 PMC6146711 29997216

[37] Obregon-Perko V, Bricker KM, Mensah G, Uddin F, Kumar MR, Fray EJ, et al. Simian-human immunodeficiency virus SHIV.C.CH505 persistence in art-suppressed infant macaques is characterized by elevated shiv RNA in the gut and a high abundance of intact SHIV DNA in naive CD4(+) T cells. J Virol. 2020;95(2):10–128. 10.1128/JVI.01669-20 PMC7944446 33087463

[38] Brenchley JM, Price DA, Schacker TW, Asher TE, Silvestri G, Rao S, et al. Microbial translocation is a cause of systemic immune activation in chronic HIV infection. Nat Med. 2006;12(12):1365–71. 17115046 10.1038/nm1511

[39] Steele AK, Lee EJ, Vestal B, Hecht D, Dong Z, Rapaport E, et al. Contribution of intestinal barrier damage, microbial translocation and HIV-1 infection status to an inflammaging signature. PLoS ONE. 2014;9(5): e97171. 24819230 10.1371/journal.pone.0097171 PMC4018269

[40] Kourtis AP, Ibegbu CC, Wiener J, King CC, Tegha G, Kamwendo D, et al. Role of intestinal mucosal integrity in HIV transmission to infants through breast-feeding: the BAN study. J Infect Dis. 2013;208(4):653–61. 23687226 10.1093/infdis/jit221 PMC3719904

[41] Zevin AS, McKinnon L, Burgener A, Klatt NR. Microbial translocation and microbiome dysbiosis in HIV-associated immune activation. Curr Opin HIV AIDS. 2016;11(2):182–90. 26679414 10.1097/COH.0000000000000234 PMC4752849

[42] Ling Z, Jin C, Xie T, Cheng Y, Li L, Wu N. Alterations in the fecal microbiota of patients with HIV-1 infection: an observational study in a Chinese population. Sci Rep. 2016;6(1):30673. 27477587 10.1038/srep30673 PMC4967929

[43] Li S, Yang X, Moog C, Wu H, Su B, Zhang T. Neglected mycobiome in HIV infection: alterations, common fungal diseases and antifungal immunity. Front Immunol. 2022. 10.3389/fimmu.2022.1015775. 36439143 10.3389/fimmu.2022.1015775 PMC9684632

[44] Hager CL, Ghannoum MA. The mycobiome in HIV. Curr Opin HIV AIDS. 2018;13(1):69–72. 29028668 10.1097/COH.0000000000000432 PMC5805152

[45] Monaco CL, Gootenberg DB, Zhao G, Handley SA, Ghebremichael MS, Lim ES, et al. Altered virome and bacterial microbiome in human immunodeficiency virus-associated acquired immunodeficiency syndrome. Cell Host Microbe. 2016;19(3):311–22. 26962942 10.1016/j.chom.2016.02.011 PMC4821831

[46] Stern J, Miller G, Li X, Saxena D. Virome and bacteriome: two sides of the same coin. Curr Opin Virol. 2019;37:37–43. 31177014 10.1016/j.coviro.2019.05.007 PMC6768692

[47] Rosel-Pech C, Pinto-Cardoso S, Chávez-Torres M, Montufar N, Osuna-Padilla I, Ávila-Ríos S, et al. Distinct fecal microbial signatures are linked to sex and chronic immune activation in pediatric HIV infection. Front Immunol. 2023. 10.3389/fimmu.2023.1244473. 37711620 10.3389/fimmu.2023.1244473 PMC10497879

[48] Sessa L, Reddel S, Manno E, Quagliariello A, Cotugno N, Del Chierico F, et al. Distinct gut microbiota profile in antiretroviral therapy-treated perinatally HIV-infected patients associated with cardiac and inflammatory biomarkers. AIDS. 2019;33(6):1001–11. 30946154 10.1097/QAD.0000000000002131

[49] Kaur US, Shet A, Rajnala N, Gopalan BP, Moar P, et al. High abundance of genus prevotella in the gut of perinatally HIV-infected children is associated with IP-10 levels despite therapy. Sci Rep. 2018;8(1):17679. 30518941 10.1038/s41598-018-35877-4 PMC6281660

[50] Flygel TT, Sovershaeva E, Claassen-Weitz S, Hjerde E, Mwaikono KS, Odland J, et al. Composition of gut microbiota of children and adolescents with perinatal human immunodeficiency virus infection taking antiretroviral therapy in Zimbabwe. J Infect Dis. 2020;221(3):483–92. 31549151 10.1093/infdis/jiz473 PMC7457326

[51] Vujkovic-Cvijin I, Sortino O, Verheij E, Sklar J, Wit FW, Kootstra NA, et al. HIV-associated gut dysbiosis is independent of sexual practice and correlates with noncommunicable diseases. Nat Commun. 2020;11(1):2448. 32415070 10.1038/s41467-020-16222-8 PMC7228978

[52] Tabung FK, Birmann BM, Epstein MM, Martínez-Maza O, Breen EC, Wu K, et al. Influence of dietary patterns on plasma soluble CD14, a surrogate marker of gut barrier dysfunction. Curr Dev Nutri. 2017;1(11): e001396. 10.3945/cdn.117.001396 PMC5867900 29595830

[53] Madhurantakam S, Lee ZJ, Naqvi A, Prasad S. Importance of IP-10 as a biomarker of host immune response: critical perspective as a target for biosensing. Curr Res Biotechnol. 2023;5: 100130.

[54] Zheng H, Na H, Yao J, Su S, Han F, Li X, et al. 16S rRNA seq-identified Corynebacterium promotes pyroptosis to aggravate diabetic foot ulcer. BMC Infect Dis. 2024;24(1):366. 38561650 10.1186/s12879-024-09235-x PMC10986075

[55] Christodoulides A, Boyadjian A, Kelesidis T. Spirochetal lipoproteins and immune evasion. Front Immunol. 2017;8:364. 28424696 10.3389/fimmu.2017.00364 PMC5372817

[56] Sainz T, Gosalbes MJ, Talavera-Rodríguez A, Jimenez-Hernandez N, Prieto L, Escosa L, et al. Effect of a nutritional intervention on the intestinal microbiota of vertically HIV-infected children: the pediabiota study. Nutrients. 2020. 10.3390/nu12072112. 32708743 10.3390/nu12072112 PMC7400861

[57] Abdugheni R, Wang W-Z, Wang Y-J, Du M-X, Liu F-L, Zhou N, et al. Metabolite profiling of human-originated Lachnospiraceae at the strain level. iMeta. 2022;1(4): e58. 38867908 10.1002/imt2.58 PMC10989990

[58] Parada Venegas D, De la Fuente MK, Landskron G, González MJ, Quera R, Dijkstra G, et al. Short chain fatty acids (SCFAs)-mediated gut epithelial and immune regulation and its relevance for inflammatory bowel diseases. Front Immunol. 2019. 10.3389/fimmu.2019.00277. 30915065 10.3389/fimmu.2019.00277 PMC6421268

[59] Borgognone A, Noguera-Julian M, Oriol B, Noël-Romas L, Ruiz-Riol M, Guillén Y, et al. Gut microbiome signatures linked to HIV-1 reservoir size and viremia control. Microbiome. 2022;10(1):59. 35410461 10.1186/s40168-022-01247-6 PMC9004083

[60] Imai K, Yamada K, Tamura M, Ochiai K, Okamoto T. Reactivation of latent HIV-1 by a wide variety of butyric acid-producing bacteria. Cell Mol Life Sci. 2012;69(15):2583–92. 22322557 10.1007/s00018-012-0936-2 PMC11114855

[61] Rocafort M, Gootenberg DB, Luévano JM, Paer JM, Hayward MR, Bramante JT, et al. HIV-associated gut microbial alterations are dependent on host and geographic context. Nat Commun. 2024;15(1):1055. 38316748 10.1038/s41467-023-44566-4 PMC10844288

[62] Yatsunenko T, Rey FE, Manary MJ, Trehan I, Dominguez-Bello MG, Contreras M, et al. Human gut microbiome viewed across age and geography. Nature. 2012;486(7402):222–7. 22699611 10.1038/nature11053 PMC3376388

[63] ClinicalinfoHIV.gov. Guidelines for the Use of Antiretroviral Agents in Pediatric HIV Infection 2023 https://clinicalinfo.hiv.gov/en/guidelines/pediatric-arv/when-initiate-therapy-antiretroviral-naive-children. Accessed 10 May 2024.

[64] Boettiger DC, Law MG, Sohn AH, Davies MA, Wools-Kaloustian K, Leroy V, et al. Temporal trends in co-trimoxazole use among children on antiretroviral therapy and the impact of co-trimoxazole on mortality rates in children without severe immunodeficiency. J Pediatric Infect Dis Soc. 2019;8(5):450–60. 30215763 10.1093/jpids/piy087 PMC6831936

[65] Bourke CD, Gough EK, Pimundu G, Shonhai A, Berejena C, Terry L, et al. Cotrimoxazole reduces systemic inflammation in HIV infection by altering the gut microbiome and immune activation. Sci Transl Med. 2019. 10.1126/scitranslmed.aav0537. 30944164 10.1126/scitranslmed.aav0537 PMC6783302

[66] Pant A, Maiti TK, Mahajan D, Das B. Human gut microbiota and drug metabolism. Microb Ecol. 2023;86(1):97–111. 35869999 10.1007/s00248-022-02081-x PMC9308113

[67] Vieira VA, Zuidewind P, Muenchhoff M, Roider J, Millar J, Clapson M, et al. Strong sex bias in elite control of paediatric HIV infection. AIDS. 2019;33(1):67–75. 30325765 10.1097/QAD.0000000000002043 PMC6750143

[68] Vieira VA, Millar J, Adland E, Muenchhoff M, Roider J, Guash CF, et al. Robust HIV-specific CD4+ and CD8+ T-cell responses distinguish elite control in adolescents living with HIV from viremic nonprogressors. AIDS. 2022;36(1):95–105. 34581306 10.1097/QAD.0000000000003078 PMC8654249

[69] Dogra SK, Kwong Chung C, Wang D, Sakwinska O, Colombo Mottaz S, Sprenger N. Nurturing the early life gut microbiome and immune maturation for long term health. Microorganisms. 2021. 10.3390/microorganisms9102110. 34683431 10.3390/microorganisms9102110 PMC8537230

[70] Kelley CF, Kitchen CM, Hunt PW, Rodriguez B, Hecht FM, Kitahata M, et al. Incomplete peripheral CD4+ cell count restoration in HIV-infected patients receiving long-term antiretroviral treatment. Clin Infect Dis. 2009;48(6):787–94. 19193107 10.1086/597093 PMC2720023

[71] Brenchley JM, Schacker TW, Ruff LE, Price DA, Taylor JH, Beilman GJ, et al. CD4+ T cell depletion during all stages of HIV disease occurs predominantly in the gastrointestinal tract. J Exp Med. 2004;200(6):749–59. 15365096 10.1084/jem.20040874 PMC2211962

[72] Chavez L, Calvanese V, Verdin E. HIV latency is established directly and early in both resting and activated primary CD4 T cells. PLoS Pathog. 2015;11(6): e1004955. 26067822 10.1371/journal.ppat.1004955 PMC4466167

[73] Sainz T, Diaz L, Rojo D, Clemente MI, Barbas C, Gosalbes MJ, et al. Targeting the gut microbiota of vertically HIV-infected children to decrease inflammation and immunoactivation: a pilot clinical trial. Nutrients. 2022. 10.3390/nu14050992. 35267967 10.3390/nu14050992 PMC8912579

[74] Falivene J, Ghiglione Y, Laufer N, Socías ME, Holgado MP, Ruiz MJ, et al. Th17 and Th17/Treg ratio at early HIV infection associate with protective HIV-specific CD8(+) T-cell responses and disease progression. Sci Rep. 2015;5:11511. 26099972 10.1038/srep11511 PMC4477236

[75] He T, Brocca-Cofano E, Policicchio BB, Sivanandham R, Gautam R, Raehtz KD, et al. Cutting edge: T Regulatory cell depletion reactivates latent simian immunodeficiency virus (SIV) in controller macaques while boosting SIV-specific T lymphocytes. J Immunol. 2016;197(12):4535–9. 27837106 10.4049/jimmunol.1601539 PMC5441309

[76] Sivanandham R, Kleinman AJ, Sette P, Brocca-Cofano E, Kilapandal Venkatraman SM, Policicchio BB, et al. Nonhuman primate testing of the impact of different regulatory T cell depletion strategies on reactivation and clearance of latent simian immunodeficiency virus. J Virol. 2020;94(19):10–128. 10.1128/JVI.00533-20 PMC7495362 32669326

[77] Hegazy AN, West NR, Stubbington MJT, Wendt E, Suijker KIM, Datsi A, et al. Circulating and tissue-resident CD4(+) T cells with reactivity to intestinal microbiota are abundant in healthy individuals and function is altered during inflammation. Gastroenterology. 2017;153(5):1320-37.e16. 28782508 10.1053/j.gastro.2017.07.047 PMC5687320

[78] Khoury G, Fromentin R, Solomon A, Hartogensis W, Killian M, Hoh R, et al. Human immunodeficiency virus persistence and t-cell activation in blood, rectal, and lymph node tissue in human immunodeficiency virus-infected individuals receiving suppressive antiretroviral therapy. J Infect Dis. 2017;215(6):911–9. 28453847 10.1093/infdis/jix039 PMC5407052

[79] Cockerham LR, Siliciano JD, Sinclair E, O’Doherty U, Palmer S, Yukl SA, et al. CD4+ and CD8+ T cell activation are associated with HIV DNA in resting CD4+ T cells. PLoS ONE. 2014;9(10): e110731. 25340755 10.1371/journal.pone.0110731 PMC4207702

[80] Muenchhoff M, Chung AW, Roider J, Dugast AS, Richardson S, Kløverpris H, et al. Distinct immunoglobulin Fc glycosylation patterns are associated with disease nonprogression and broadly neutralizing antibody responses in children with HIV infection. MSphere. 2020;5(6):10–128. 10.1128/mSphere.00880-20 PMC7763548 33361123

[81] Aggarwal H, Khan L, Chaudhary O, Kumar S, Makhdoomi MA, Singh R, et al. Alterations in B cell compartment correlate with poor neutralization response and disease progression in HIV-1 infected children. Front Immunol. 2017;8:1697. 29250072 10.3389/fimmu.2017.01697 PMC5717014

[82] Steel JH, Malatos S, Kennea N, Edwards AD, Miles L, Duggan P, et al. Bacteria and inflammatory cells in fetal membranes do not always cause preterm labor. Pediatr Res. 2005;57(3):404–11. 15659699 10.1203/01.PDR.0000153869.96337.90

[83] Amarasekara R, Jayasekara RW, Senanayake H, Dissanayake VH. Microbiome of the placenta in pre-eclampsia supports the role of bacteria in the multifactorial cause of pre-eclampsia. J Obstet Gynaecol Res. 2015;41(5):662–9. 25492799 10.1111/jog.12619

[84] Mitsou EK, Kirtzalidou E, Oikonomou I, Liosis G, Kyriacou A. Fecal microflora of Greek healthy neonates. Anaerobe. 2008;14(2):94–101. 18207437 10.1016/j.anaerobe.2007.11.002

[85] Kabeerdoss J, Ferdous S, Balamurugan R, Mechenro J, Vidya R, Santhanam S, et al. Development of the gut microbiota in southern Indian infants from birth to 6 months: a molecular analysis. J Nutr Sci. 2013;2: e18. 25191566 10.1017/jns.2013.6 PMC4153310

[86] Dominguez-Bello MG, Costello EK, Contreras M, Magris M, Hidalgo G, Fierer N, et al. Delivery mode shapes the acquisition and structure of the initial microbiota across multiple body habitats in newborns. Proc Natl Acad Sci. 2010;107(26):11971–5. 20566857 10.1073/pnas.1002601107 PMC2900693

[87] Semon AK, Keenan O, Zackular JP. Clostridioides difficile and the microbiota early in life. J Pediatric Infect Dis Soc. 2021;103:7. 10.1093/jpids/piab063 PMC8600012 34791400

[88] Bäckhed F, Roswall J, Peng Y, Feng Q, Jia H, Kovatcheva-Datchary P, et al. Dynamics and stabilization of the human gut microbiome during the first year of life. Cell Host Microbe. 2015;17(5):690–703. 25974306 10.1016/j.chom.2015.04.004

[89] Laursen MF, Bahl MI, Michaelsen KF, Licht TR. First foods and gut microbes. Front Microbiol. 2017;8:356. 28321211 10.3389/fmicb.2017.00356 PMC5337510

[90] Stewart CJ, Ajami NJ, O’Brien JL, Hutchinson DS, Smith DP, Wong MC, et al. Temporal development of the gut microbiome in early childhood from the TEDDY study. Nature. 2018;562(7728):583–8. 30356187 10.1038/s41586-018-0617-x PMC6415775

[91] Serrano-Villar S, de Lagarde M, Vázquez-Castellanos J, Vallejo A, Bernadino JI, Madrid N, et al. Effects of immunonutrition in advanced human immunodeficiency virus disease: a randomized placebo-controlled clinical trial (promaltia study). Clin Infect Dis. 2019;68(1):120–30. 29788075 10.1093/cid/ciy414

[92] Reikvam DH, Meyer-Myklestad MH, Trøseid M, Stiksrud B. Probiotics to manage inflammation in HIV infection. Curr Opin Infect Dis. 2020;33(1):34–43. 31789692 10.1097/QCO.0000000000000612

[93] Ishizaki A, Bi X, Nguyen LV, Matsuda K, Pham HV, Phan CTT, et al. Effects of short-term probiotic ingestion on immune profiles and microbial translocation among HIV-1-infected Vietnamese children. Int J Mol Sci. 2017. 10.3390/ijms18102185. 29048352 10.3390/ijms18102185 PMC5666866

[94] Trois L, Cardoso EM, Miura E. Use of probiotics in HIV-infected children: a randomized double-blind controlled study. J Trop Pediatr. 2008;54(1):19–24. 17878180 10.1093/tropej/fmm066

[95] Gautam N, Dayal R, Agarwal D, Kumar R, Singh TP, Hussain T, et al. Role of multivitamins, micronutrients and probiotics supplementation in management of HIV infected children. Indian J Pediatr. 2014;81(12):1315–20. 24760382 10.1007/s12098-014-1407-6

[96] Athiyyah AF, Brahmantya H, Dwiastuti S, Darma A, Puspitasari D, Husada D, et al. Effect of lactobacillus plantarum IS-10506 on blood lipopolysaccharide level and immune response in HIV-infected children. Iran J Microbiol. 2019;11(2):137–44. 31341568 PMC6635306

[97] Cunningham-Rundles S, Ahrné S, Bengmark S, Johann-Liang R, Marshall F, Metakis L, et al. Probiotics and immune response. Am J Gastroenterol. 2000;95(1 Suppl):S22–5. 10634225 10.1016/s0002-9270(99)00813-8

[98] O’Brien CE, Meier AK, Cernioglo K, Mitchell RD, Casaburi G, Frese SA, et al. Early probiotic supplementation with B infantis in breastfed infants leads to persistent colonization at 1 year. Pediatr Res. 2022;91(3):627–36. 33762689 10.1038/s41390-020-01350-0 PMC8460680

[99] Serrano-Villar S, Talavera-Rodríguez A, Gosalbes MJ, Madrid N, Pérez-Molina JA, Elliott RJ, et al. Fecal microbiota transplantation in HIV: a pilot placebo-controlled study. Nat Commun. 2021;12(1):1139. 33602945 10.1038/s41467-021-21472-1 PMC7892558

[100] Utay NS, Monczor AN, Somasunderam A, Lupo S, Jiang ZD, Alexander AS, et al. Evaluation of six weekly oral fecal microbiota transplants in people with HIV. Pathog Immun. 2020;5(1):364–81. 33501400 10.20411/pai.v5i1.388 PMC7815055

[101] Karolewska-Bochenek K, Grzesiowski P, Banaszkiewicz A, Gawronska A, Kotowska M, Dziekiewicz M, et al. A two-week fecal microbiota transplantation course in pediatric patients with inflammatory bowel disease. Adv Exp Med Biol. 2018;1047:81–7. 29151253 10.1007/5584_2017_123

[102] Zou B, Liu S-X, Li X-S, He J-Y, Dong C, Ruan M-L, et al. Long-term safety and efficacy of fecal microbiota transplantation in 74 children: a single-center retrospective study. Front Pediatr. 2022. 10.3389/fped.2022.964154. 36304525 10.3389/fped.2022.964154 PMC9595213

[103] Nicholson MR, Mitchell PD, Alexander E, Ballal S, Bartlett M, Becker P, et al. Efficacy of fecal microbiota transplantation for clostridium difficile infection in children. Clin Gastroenterol Hepatol. 2020;18(3):612-9.e1. 31009795 10.1016/j.cgh.2019.04.037 PMC7549313

